# Assessing the Effect of Oxytetracycline on the Selection of Resistant *Escherichia coli* in Treated and Untreated Broiler Chickens

**DOI:** 10.3390/antibiotics12121652

**Published:** 2023-11-23

**Authors:** Ekaterina Pokrant, María Belén Vargas, María José Navarrete, Karina Yévenes, Lina Trincado, Paula Cortés, Aldo Maddaleno, Lisette Lapierre, Javiera Cornejo

**Affiliations:** 1 Laboratory of Food Safety, Department of Preventive Animal Medicine, Faculty of Veterinary and Animal Sciences, University of Chile, Santiago 8820808, Chile; katiavalerievna@uchile.cl (E.P.); maria.vargas.s@ug.uchile.cl (M.B.V.); maria.navarrete.g@ug.uchile.cl (M.J.N.); kyevenes@ug.uchile.cl (K.Y.); 2 Doctorate Program of Forestry, Agriculture, and Veterinary Sciences (DCSAV), University of Chile, Av. Santa Rosa 11315, La Pintana, Santiago 8820808, Chile; lina.trincado@ug.uchile.cl; 3 Laboratory of Veterinary Pharmacology (FARMAVET), Faculty of Veterinary and Animal Sciences, University of Chile, Santiago 8820808, Chile; paula.cortes@ug.uchile.cl (P.C.); amaddaleno@veterinaria.uchile.cl (A.M.); 4 Laboratory of Bacterial Pathogens Diagnostic and Antimicrobial Resistance, Department of Preventive Animal Medicine, Faculty of Veterinary and Animal Sciences, University of Chile, Santiago 8820808, Chile

**Keywords:** *Escherichia coli*, antibiotic resistance, droppings, broiler litter, oxytetracycline, resistance genes

## Abstract

Oxytetracycline (OTC) is administered in the poultry industry for the treatment of digestive and respiratory diseases. The use of OTC may contribute to the selection of resistant bacteria in the gastrointestinal tract of birds or in the environment. To determine the effect of OTC on the selection of resistant *Escherichia coli* strains post-treatment, bacteria were isolated from droppings and litter sampled from untreated and treated birds. Bacterial susceptibility to tetracyclines was determined by the Kirby–Bauer test. A total of 187 resistant isolates were analyzed for the presence of *tet*(A), (B), (C), (D), (E), and (M) genes by PCR. Fifty-four strains were analyzed by PFGE for subtyping. The proportion of tetracycline-resistant *E. coli* strains isolated was 42.88%. The susceptibility of the strains was treatment-dependent. A high clonal diversity was observed, with the *tet*(A) gene being the most prevalent, followed by *tet*(C). Even at therapeutic doses, there is selection pressure on resistant *E. coli* strains. The most prevalent resistance genes were *tet*(A) and *tet*(C), which could suggest that one of the main mechanisms of resistance of *E. coli* to tetracyclines is through active efflux pumps.

## 1. Introduction

One of the most important side effects of using antimicrobials in productive animals is the selection of resistant bacteria. Efforts have been made to control the improper or excessive use of antimicrobials, highlighting the global action plan on antimicrobial resistance adopted at the World Health Assembly in May 2015, where different objectives related to this issue were established [1]. The use of tetracyclines has led to the emergence of resistant bacterial variants, in particular those containing *tet* genes, which are generally associated with mobile genetic elements or conjugative transposons [2,3,4]. These elements code for different resistance mechanisms, such as efflux pumps, ribosomal protection, enzymatic inactivation, and mutations, such as the one described in the 30S ribosomal subunit. In Gram-negative bacteria, the efflux pump systems are encoded by *tet* genes, mostly by *tet*(A)*, tet*(B), *tet*(C), and *tet*(E), while the *tet*(M) gene, which is one of the most studied determinants, encodes for ribosomal protection [5].

Antibiotic resistance genes (ARGs) are currently considered to be emerging contaminants because they have been detected throughout the environment, including soils, river sediments, watercourses, and wastewater [6,7]. The presence of ARGs in different environments is a risk because it confers selection pressure on pathogenic and commensal strains at the genetic level, leading to the emergence of antimicrobial resistance. The genes can be acquired by other pathogenic bacteria that affect humans and animals, which has a socio-economic impact due to increased treatment costs and production losses due to the spread of ARGs [8,9].

*Escherichia coli* is a Gram-negative bacillus that is part of the normal intestinal microbiota of animals and humans. However, there are some pathogenic strains that can cause fatal diseases in the host [10]. These bacteria are used as indicator bacteria for antimicrobial resistance levels in different productive species, as they have been described as a reservoir of resistance genes, which could be transmitted to pathogenic and zoonotic bacteria [10,11,12,13]. Antimicrobial use increases the risk of antimicrobial resistance in *E. coli* in pigs. However, there is little information concerning the impact of dose or concentration and or the effects of antimicrobial use over time [14]. Chantziaras et al. [15] evaluated the correlation between antibiotic use and the prevalence of *E. coli* strains isolated from pigs, poultry, and cattle. Their results indicated that there is a correlation between the use of specific antimicrobials and the level of resistance of these microorganisms. They concluded that more detailed data collection and harmonization are needed due to data restrictions in their study. Although it has been previously observed that oral administration of tetracycline does not induce significant changes in the cecal bacterial community of chickens, a relationship between its use as a growth promotor and an increase in the population of tetracycline-resistant *E. coli* harboring *tet*(A) or *tet*(B) has been shown. However, the effect of therapeutic doses of oxytetracycline (OTC) on the selection of tetracycline-resistant *E. coli* under controlled conditions has not been demonstrated [15].

Da Costa et al. [16] investigated variations in the prevalence of antibiotic resistance in *E. coli* isolated from the fecal matter of broilers raised with three different commercial antimicrobial treatments administered through their water supply. After the administration of different antibiotics, resistance grew quickly. The sharp rise in antimicrobial resistance rates following drug administration was a direct result of the formation of new antimicrobial resistance patterns rather than the amplification of previously resistant organisms [16].

Herrero-Fresno et al. [17] determined that the intestinal microbiota of apramycin-treated pigs showed resistance selection from treatment, resulting in noticeably greater counts of resistant strains than untreated pigs [17]. More recently, Das et al. [18] analyzed the incidence and distribution of oxytetracycline- and ciprofloxacin-resistant *E. coli* isolated from live broilers and the farm environment. They determined that 100% of commensal *E. coli* strains isolated from chickens and the environment were resistant to tetracyclines, with the majority harboring the *tet*(A) gene [18].

In an in vivo study, Shah et al. [19] determined that the use of antimicrobials such as oxytetracycline as growth promoters in the diet has no detrimental effect on beneficial bacteria, but an alteration in the growth of harmful bacteria was observed [19]. However, there are no known controlled studies that determine the effect of OTC administration, at therapeutic doses, in broilers on the selection of post-treatment resistant strains of *E. coli*. The objective of this study was to determine the presence of *E. coli* isolates not susceptible to OTC and resistance genes in broiler droppings and litter from treated and untreated birds and to determine if there is a relationship between treatment and the selection of resistant bacteria. The purpose of this study focused on generating further scientific knowledge regarding whether the use of OTC at therapeutic doses predisposed to the selection of resistant *E. coli* in a controlled environment by determining phenotypic and genotypic resistance in droppings and poultry litter, as these by-products could be a potential source of reservoir and future dissemination of ARGs into the environment.

## 2. Results

### 2.1. Isolation and Confirmation of E. coli

*E. coli* were isolated from samples collected the day before treatment to determine a base resistance profile. *E. coli* was only isolated from manure samples. *E. coli* isolates were confirmed by biochemical tests. No microorganisms were observed in the litter samples prior to treatment.

**Figure 1:**
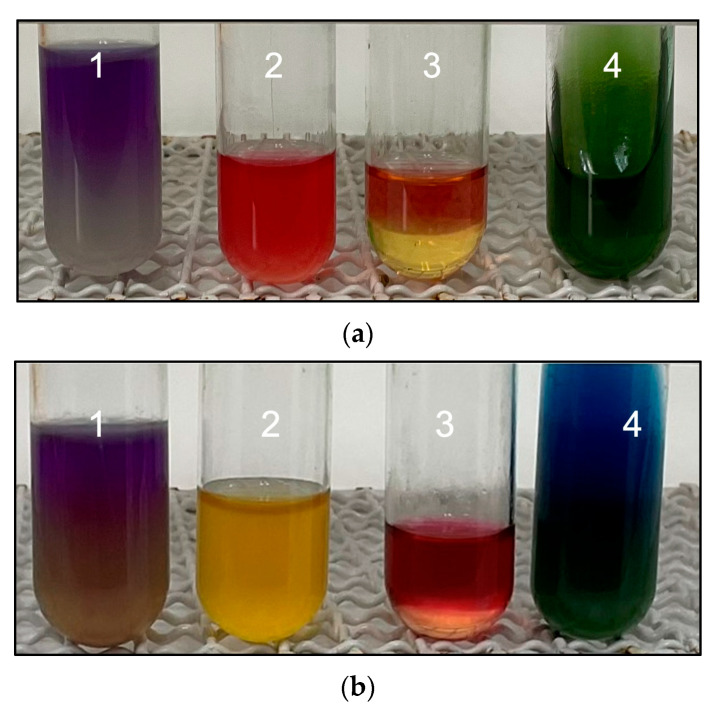
Representative images of IMViC for the confirmation of *E. coli* strains: (**a**) positive reaction to *E. coli*. [1: MIO agar (+); 2: methyl red (+); 3: Voges–Proskauer broth (−); 4: citrate agar (−)] and (**b**) negative reaction to *E. coli*. [1: MIO agar (−); 2: methyl red (−); 3: Voges–Proskauer broth (+); 4: citrate agar (+)].

Post-treatment, *E. coli* colonies were isolated and confirmed by biochemical tests and PCR. Six manure samples and six litter samples were analyzed for each experimental group on days 1, 7, 14, and 21 post-treatment, and six litter samples for each experimental group on days 7 and 14 post-slaughter. Five typical colonies on MacConkey agar were obtained from each sample and confirmed by the IMViC test: positive for ornithine, motility, and methyl red and negative for Voges–Proskauer and citrate (Figure 1).

### 2.2. Determination of Antimicrobial Susceptibility

Susceptible and resistant strains were detected at all sample points in both cloacal and litter samples. Strains with intermediate sensitivity were categorized as resistant. Non-susceptible strains were found in both the treated and untreated groups, with the highest percentage in the treatment group on day one post-treatment in litter and droppings (Table 1 and Table 2).

The OTC treatment in poultry had a significant effect on tetracycline *E. coli* resistance isolates of droppings, where the chi-squared test showed an association between the treated group and *E. coli* resistance (Table 1). On another hand, McNemar’s test indicated an increase in *E. coli* resistance from the same individual’s droppings pre-treatment relative to post-treatment (*p* < 0.05).

In relation to the isolates detected in poultry litters, there were significant differences between the treated and untreated groups, showing that the treatment affected the resistance of *E. coli* to tetracyclines isolated from litter (Table 2).

The emergence of tetracycline resistance among commensal organisms such as *E. coli* may result in a non-response to antibiotic therapy, as these microorganisms can be reservoirs of ARGs, which can be transferred to avian pathogens, increasing mortality and thus economic losses at the production level.

### 2.3. Detection of Resistance Genes

**Figure 2:**
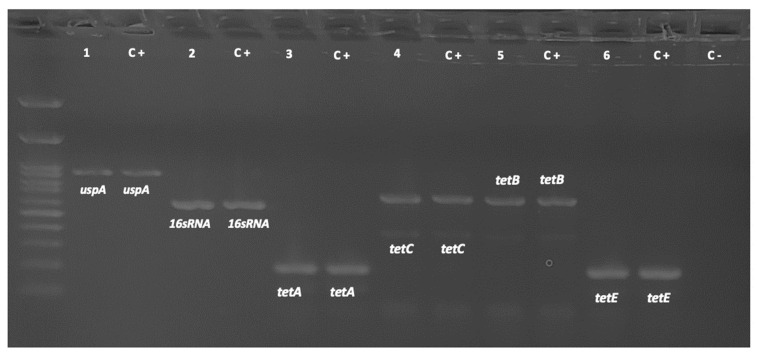
Representative image of the PCR products observed on 2% agarose gel. *tet*(A), 210 bp; *tet*(B), 659 bp; *tet*(C), 418 bp; *tet*(E), 278 bp; *uspA*, 884 bp; and *16s*RNA, 585 bp. Lanes 1 to 6 correspond to target gene amplicons, and lane C+ corresponds to the positive control for each gene.

The presence of *tet*(A), *tet*(B), *tet*(C), *tet*(E), and *tet*(M) genes in the resistant *E. coli* strains was determined by conventional PCR. Figure 2 shows the amplification products for the detected genes.

The most prevalent resistance gene was *tet*(A), followed by *tet*(C), *tet*(B), *tet*(M), and *tet*(E).

Molecularly, *tet* genes are resistance determinants, which encode inactivation mechanisms comprising the efflux pump system and ribosomal protection. The *tet*(A) gene was one of the most frequently detected genes in this study, which is consistent with the literature. Studies describe that this gene is one of the most frequently detected genes in both Gram-positive and Gram-negative bacteria and encodes for efflux pump systems that contribute to tetracycline resistance by constantly reducing the antibiotic concentration inside the cell [2]. Thus, this gene can be a potential risk of dissemination within the production chain and contribute to antimicrobial resistance.

### 2.4. Isolate Subtyping

Fifty-four *E. coli* strains isolated pre-treatment and on day 1 post-treatment were analyzed by PFGE genotyping. The criteria for isolate selection for PFGE are based on the fact that OTC at therapeutic doses immediately eliminates sensitive bacteria and selects for resistant strains; when there is a predominance of one clone, it would continue to spread during treatment. The PFGE technique was also used to observe whether there was a predominance of a single clone or multiple clones.

**Figure 3:**
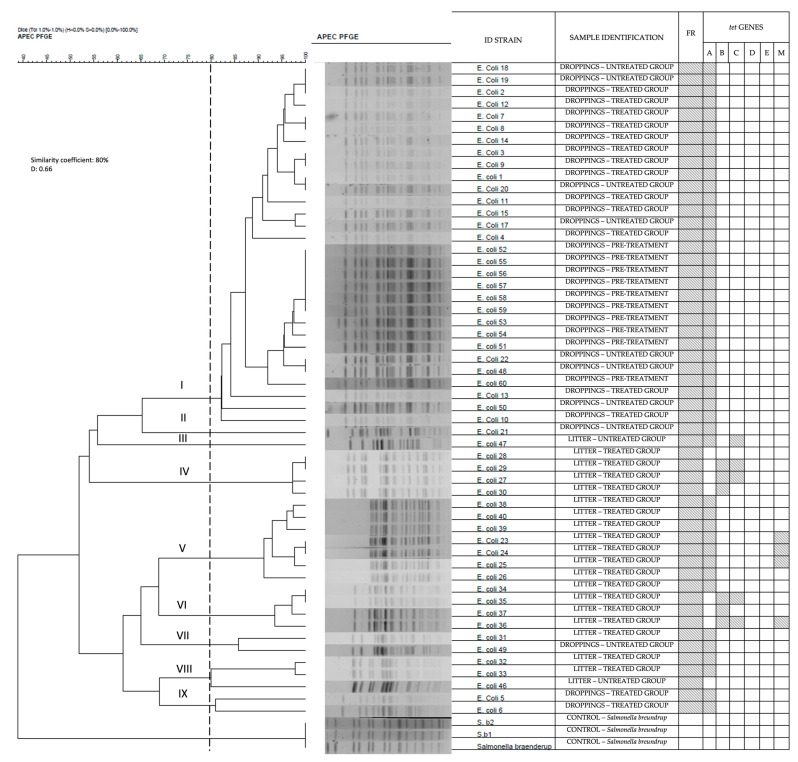
Dendrogram of the cluster analysis of *E. coli* strains generated by Gel Compar II software, version 5.10, using the unweighted pair group arithmetic mean method (UPGMA), with 1% tolerance. The resistance genes detected in each strain are marked with a color. The analyzed strains were isolated from the samples prior to the start of treatment and the first sampling point (day 1 post-treatment). From I to IX, corresponds to the different clusters. Shaded areas indicate positivity. D: Simpson diversity index.

A phylogenetic analysis was performed using the PFGE pulsotypes and was compared to the antibiotic resistance profile (Figure 3). The PFGE pulsotypes were classified into 42 types. The Simpson diversity index was 0.66, which means that the isolated *E. coli* was highly diverse. The phylogenetic dendrogram classified the *E. coli* strains into nine clusters. The samples from droppings were grouped mainly in clusters I, II, VII, and IX, while those from the litter were grouped in clusters III, IV, V, VI, and VIII. *tet*(A) genes were found in almost all isolates, both before and after treatment. *tet*(D) and *tet*(E) genes were not found. Isolates from clusters III, IV, V, and VI contained *tet*(B), *tet*(C), and *tet*(M) genes and were isolated exclusively from litter samples. Only eight isolates, also all litter samples, were negative for the presence of the *tet* genes.

## 3. Discussion

In this study, the highest prevalence of resistant isolates came from droppings and litter samples from OTC-treated poultry. Although it was not possible to statistically correlate the concentrations with the selection of resistant bacteria, a higher prevalence of non-susceptible *E. coli* was determined in the first sampling point, where the concentrations in both matrices were significantly higher than in the subsequent sampling. Therefore, it is inferred that the isolated *E. coli* population in the first point underwent a higher selection pressure by the excreted antimicrobial, where the concentrations of OTC and its epimer detected were 22,742 µg kg^−1^ in litter and 2,087 µg kg^−1^ in droppings of treated animals [20].

High concentrations of antimicrobials have been shown to produce selection pressure on resistant microbiota [21]. Berge et al. [22], who studied bacterial resistance patterns in *E. coli* from cattle fecal samples after a single dose of florfenicol, observed an increase in the number of resistant *E. coli* isolates [22]. Similarly, Fairchild et al. [23] investigated the effects of tetracycline administration on commensal bacteria from commercial poultry and found that *Enterococcus* spp. and *E. coli* were resistant to tetracyclines with 32.2% harboring *tet*(A) and 30.5% containing *tet*(B) resistance genes [23].

Low residue concentrations, even below the detection limit (LOD), may also be associated with the presence of resistant microorganisms [24,25,26]. Resistance resulting from even low doses of antibiotics is of worldwide concern since, currently, OTC is not only used for the treatment of productive animals but also in the poultry industry at subtherapeutic doses through feed to promote growth. Use to promote growth is still allowed in some countries, such as Brazil and China [27].

The presence of pre-treatment *E. coli* in manure samples can be attributed to the fact that this bacterium is a commensal microorganism, so birds are colonized during their first days of life; however, the detection of resistant strains could be due to selection pressure contributing to the emergence of resistance genes and their potential dissemination through these production systems. On the other hand, prior to treatment, the wood shavings used for litter were sampled, and according to the analyses, no *E. coli* was isolated from this product. Therefore, and considering that the wood shavings have a very low water activity, we can assure that there was no external contamination from this litter component.

The phenotype for tetracycline resistance matched the genotypic resistance, determined by *tet* gene positivity, in almost all isolates in 75 non-susceptible *E. coli* isolates from droppings at days 1, 7, 14, and 21 post-treatment and 97 *E. coli* isolates from litter samples at days 1, 14, 21, 29, and 36 post-treatment. Most of the genes present in the isolates from droppings and litter were *tet*(A), followed by *tet*(C), *tet*(B), *tet*(M), and *tet*(E). None of the isolates harbored *tet*(D). Only 17 resistant isolates had none of the resistance genes analyzed; therefore, the resistance of these strains could be mediated by a different gene. This discrepancy between phenotypic and genotypic resistance could be due to mutations in resistance genes. In a previous study, where this difference between genotypic versus phenotypic evidence of rifampicin resistance in *Mycobacterium tuberculosis* was observed, mutations in the resistance regions were determined [28].

Moreover, many tetracycline resistance genes have been described. The *tet* genes encode for different resistance mechanisms, such as efflux pumps, ribosomal protection, enzymatic modification, and other unknown mechanisms [2]. Therefore, it is possible that a *tet* gene, other than *tet*(A), (B), (C), (D), (E), and (M), may be mediating the resistance of isolates showing phenotypic resistance by disc diffusion test. It is also possible to attribute this resistance to a new, undescribed gene. Davis et al. [29] observed a new aminoglycoside resistance gene, *rmtE*, that belongs to the 16S ribosomal RNA methylase gene family [29].

In this study, a high clonal diversity was observed that could be the result of the adaptability of *E. coli* strains. Resistant *E. coli* isolates are highly represented by these resistance genes. This finding is consistent with the results of previous studies, where the most prevalent genes reported have been *tet*(A) and *tet*(B) [30,31]. In a current study, Sreejith et al. [32] determined through susceptibility analysis that 77% of the *E. coli* isolates were tetracycline resistant, where 85.18% of the isolates had *tet*(A) genes and 22.22% had *tet*(B) genes [32]. The high prevalence of *tet*(B) could be explained by the gene’s ability to reside on highly mobile genetic elements that efficiently transfer the gene between bacteria, like plasmids. The ability of *tet*(A) to spread freely and rapidly in farm animals and in the environment has also been reported [33].

In this study, the fourth most prevalent gene was *tet*(M), which was found in 14% of the isolates. Other studies have determined a prevalence of *tet*(M) between 5% and 13% in *E. coli* strains [31,34,35].

In this study, PFGE grouped the isolates containing the *tet*(B) gene in clusters IV and VI, which came exclusively from the litter. This could be explained by the selective pressure that occurs in the litter, where *tet*(B) was detected in isolates shortly after treatment. Sreejith et al. [32] found that the presence of antibiotics in feed and in the farm environment can help *tet*(A) and *tet*(B) persist in the microbiome for a long time. The presence of antibiotics, even at low concentrations, ensures the persistence of these resistant genes, which can be expressed dominantly in the microbial community [32]. This aspect becomes relevant as these bacteria could be a reservoir of resistance genes, which can be transmitted to other microorganisms [36].

Previous studies have shown the effects of the use of antibiotics at subtherapeutic doses on the selection of resistant bacteria [24,25]. We found an association between the therapeutic treatment given to the birds and the presence of resistant bacteria. Others have shown the presence of resistant bacteria and resistance genes is due to the strong selective pressure provided by the presence of antibiotic treatment [37]. Our results show the use of antimicrobials at therapeutic doses in poultry production can lead to the selection and persistence of resistant *E. coli* strains, which can be a risk to both human and animal health. It is important to highlight that resistant bacteria with transferable resistance genes were detected up to 36 days post-treatment in the chicken litter. We consider that one of the limitations of our study is that only one concentration of the antimicrobial was studied, so we cannot conclude similarities with respect to the lower doses under the same conditions; however, this study lays the foundation for further research in this area and provides scientific information that supports the need to monitor and control the antimicrobials used in the animal production industry.

The results provide scientific information that supports the growing concern about the use of antimicrobials in animal production and the constant worldwide effort to reduce the use of these veterinary drugs to preserve their efficacy since different antimicrobials are critical in veterinary and human medicine. For this reason, the constant monitoring and responsible use of these veterinary drugs is essential.

## 4. Materials and Methods

### 4.1. Experimental Animals

Commercial male broilers from Ross 308 genetic line (Ross^®^, Aviagen Inc., Huntsville, AL, USA), which is characterized by high yields, strong disease resistance, and weight gain, were raised from birth in an experimental unit specially designed to carry out this study. In this experiment, the birds were kept in pens of 1.5 m^2^ surface area with clean shavings that later became part of the birds’ litter. Temperature (25 ± 5 °C), humidity (50–60%), and ventilation were controlled in the unit. The animals were kept with ad libitum access to water and non-medicated feed. This study was approved by the Institutional Animal Care and Use Committee (CICUA by its Spanish acronym) through the certificate No. 18187-VET-UCH-E1. Handling and euthanasia were based on Directive 2010/63/EU and the AVMA Guidelines for the Euthanasia of Animals: 2020 Edition [38,39].

The birds were separated into a treated group and an untreated group. Group A (treatment) was 6 birds treated orally with a pharmaceutical formulation containing OTC at 10% at a therapeutical dose of 80 mg kg^−1^ for 10 consecutive days. The antibiotic was administered by orogastric tube to ensure the complete intake of the dose for each bird. Group B (non-treatment) had 6 untreated birds under the same conditions. To avoid cross-contamination, we followed the biosafety measures established by the CONICYT Biosafety Standards Manual [40], along with the biosafety standards instituted by the FAVET Biosafety Committee.

### 4.2. Sampling Collection

Cloacal and litter samples were collected at 5 sampling points: before treatment and on days 1, 7, 14, and 21 post-treatment. In addition, litter samples were analyzed one and two weeks after the birds were slaughtered (corresponding to days 29 and 36 post-treatment). Cloacal samples were collected from each bird with sterile cotton swabs and were stored in sterile polypropylene tubes. Ten grams of litter were collected from each group and were stored in sterile plastic bags. All samples were processed immediately.

### 4.3. E. coli Identification and Isolation

One gram of litter was homogenized with 9 mL peptone water for *E. coli* isolation. The cloacal samples were homogenized with 4.5 mL buffered peptone water (Huankai Microbial^®^, Guangzhou, China). Three loops of the enriched sample were streaked on MacConkey agar (OXOID^®^, Hants, UK) plates and incubated at 37 °C for 24 h. Triplicates of each sample were performed. After incubation, five typical colonies per plate were collected, and biochemical identification was performed to confirm *E. coli* colonies using IMViC test [41,42]. In addition, PCR analysis was carried out for *uspA* gene detection for confirmation of *E. coli* identity [43]. Confirmed isolates were stored at −20 °C in 20% glycerol.

### 4.4. Antimicrobial Susceptibility Testing

All confirmed *E. coli* isolates were analyzed by the Kirby–Bauer disk diffusion method, which was performed using antimicrobial susceptibility test discs in Mueller–Hinton agar (Sigma Aldrich^®^, Saint Louis, MI, USA) according to the recommendation of the Clinical and Laboratory Standards Institute (CLSI). First, a suspension of fresh, pure culture was prepared, and the turbidity of the bacterial suspension was adjusted with 0.85% saline solution until an OD_600nm_ between 0.08 and 0.1 was reached, which is equivalent to 0.5 McFarland turbidity. The adjusted suspension was inoculated into Mueller–Hinton agar plates, and tetracycline disks (30 µg) (OXOID^®^, Hants, UK) were positioned over the inoculated plate. The plates were incubated inverted at 35 °C for 16–18 h, and the inhibition halos were measured. The diameter of the inhibition zone of each disc was compared with the interpretation criteria of the Clinical and Laboratory Standards Institute guidelines. *E. coli* ATCC 25922 was used as a quality control [44].

### 4.5. PCR Detection of Tet Genes

The presence of resistance *tet* genes was determined by conventional polymerase chain reaction or PCR. After *E*. *coli* identification and isolation, template DNA was extracted from MacConkey agar plates using heat treatment [45] and was quantified by spectrophotometry (NANO-400 microspectrophotometer, Hangzhou Allsheng instruments Co., Hangzhou, China). Samples that exhibited an absorbance ratio of 260/280 nm close to the optimal range (1.8–2.0) were analyzed by PCR. The genes analyzed were *tet*(A), *tet*(B), *tet(*C), *tet*(D), *tet*(E), and *tet*(M) [46]; the 16S rRNA gene was included for confirmation of DNA presence [47] (Table 3).

For the identification of the genes *tet*(A), *tet*(B), *tet*(C), *tet*(D), and *tet*(E), PCR multiplex reactions were performed using GoTaq^®^ Green Master Mix following manufacturer’s instructions (Promega, Madison, WI, USA). For a 25 µL reaction, a mixture of 12.5 µL of GoTaq^®^ Green Master Mix, 1 µL of nuclease-free water, 1 µL of primer *tet*(A)-F, 1 µL of primer *tet*(A)-R, 1 µL of primer *tet*(B)-F, 1 µL of primer *tet*(B)-R, 1 µL of primer *tet*(C)-F, 1 µL of primer *tet*(C)-R, 1 µL of primer *tet*(D)-F, 1 µL of primer *tet*(D)-R, 1 µL of primer *tet*(E)-F, 1 µL of primer *tet*(E)-R, and 1 µL of DNA sample was prepared.

For the identification of the genes *tet*(M), PCR single reactions were performed. For a 25 µL reaction, a mixture of 12.5 µL of GoTaq^®^ Green Master Mix, 9 µL of nuclease-free water, 1.25 µL of primer *tet*(M)-F, 1.25 µL of primer *tet*(M)-R, and 1 µL of DNA sample was prepared.

Nuclease-free water was used as a negative control. On the other hand, previously sequenced DNA from strains positive for the genes studied was used as a positive control.

The PCR protocol included an initial denaturation step at 94 °C for 5 min, followed by 35 cycles of denaturation (94 °C for 1 min), annealing (*tet*: 54 °C for 1 min; *uspA* and 16S rRNA: 58 °C for 1 min), and elongation (72 °C for 1 min), with a final extension step at 72 °C for 10 min. Five µL of the PCR product was visualized on electrophoresis gels (2% (*w*/*v*) agarose in 1 × TAE buffer), previously stained with SafeView Plus (Fermelo Biotec, Santiago, Chile). The bands were visualized by ultraviolet transillumination, and the sizes of the PCR products were determined using the 100 base pair (bp) size scale (Maestrogen Hsinchu, Taiwan). Isolated strains previously sequenced for the genes studied were used as positive controls.

### 4.6. Pulsed-Field Gel Electrophoresis

Genetic relatedness among the tetracycline-resistant *E. coli* isolates was established from their *XbaI*-digested chromosomal DNA fragments. The clonality of 54 *E. coli* strains isolated from litter and droppings samples was determined by pulsed-field gel electrophoresis (PFGE) subtyping. Resistant strains from sampling point 0 (pre-treatment day) and post-treatment day 1 from the two experimental groups were analyzed. The first sampling point was considered the most representative as it was carried out immediately after the end of treatment.

The PFGE technique was performed according to the Centers for Disease Control and Prevention (CDC) using the Standard Operating Procedure for PulseNet PFGE of *E. coli* non-O157 (STEC) [48] with minor modifications. Bacterial isolates were suspended in cell suspension buffer (CSB; 100 mM Tris:100 mM EDTA, pH 8.0) at a wavelength of 420 nm (OD = 0.4). The bacterial suspension was mixed with proteinase K (20 mg/mL) and 1% melted SeaKem Gold agarose for cell lysis and carefully transferred into plug molds, which were then cooled to 4 °C. The solid plugs were washed and digested overnight with restriction enzyme *XbaI* (Thermo-Fisher Scientific, Waltham, MA, USA). After restriction digestion, electrophoresis was performed with the CHEF-DR III (Bio-Rad) system using 1% pulsed-field certified agarose (Bio-Rad, Hercules, CA, USA) in 0.5 X TBE (45 mM Tris-HCl, 45 mM boric acid, 1 mM EDTA). The electrophoresis conditions were as follows: initial switch time 2.16 s, final switch time 54.7 s, run time 20 h, included angle 120°, gradient 6 V/cm, and temperature 14 °C. The gel was stained for 30 min with Gelred^®^, and the fingerprinting profile was observed by an illuminated UV wave to the gel. DNA bands on agarose gels were pictured and saved in TIFF format. DNA band profiles were analyzed by GelCompar II Software v 5.1 (Applied Maths, Sint-Martens-Latem, Belgium).

The PFGE profiles were compared using a Dice similarity coefficient and UPGMA analysis to create the dendrogram; a band position tolerance of 1% was used. A cutoff point of 80% was used to analyze genetic relatedness and establish genetic patterns. Genetic diversity was quantified using the Simpson diversity index.

### 4.7. Data Analysis

To determine the effect of OTC treatment on tetracycline *E. coli* resistance from the same individual’s droppings, the McNemar’s test was performed, where the classification criteria were antimicrobial susceptibility (susceptible and non-susceptible) and condition (pre-treatment and post-treatment). Moreover, to determine the effect of OTC treatment on tetracycline *E. coli* resistance from litter and droppings between groups, the chi-squared test was performed. Frequencies corresponded to the percentages of non-susceptible and susceptible isolates. Rstudio^®^ V0.99.903 was used for analysis. A statistically significant difference was considered when the *p*-value < 0.05. For the genotypic resistance analysis, a genetic profile was performed using the results of the conventional PCR for the design of the different genetic patterns obtained.

## 5. Conclusions

The highest proportion of non-susceptible *E. coli* isolates (resistant and of intermediate sensitivity) to tetracyclines was detected in dropping and litter samples from the group treated with OTC. Therefore, even at therapeutic doses, there is selection pressure on *E. coli* strains resistant to tetracyclines isolated from litter. The *tet*(A) and *tet*(C) genes were most frequently identified; therefore, we conclude that the main mechanism of resistance in the *E. coli* isolates from our study was mediated by active efflux pumps. The PFGE analysis showed high clonal diversity; however, some clonal strains were isolated from the excreta of both the treated and untreated group, suggesting a local spread of these microorganisms. These results lay the foundation for future controlled studies considering different antimicrobial doses and distance between experimental groups to determine the dissemination and persistence of resistant bacteria and resistance determinants in both animals and the environment.

## Figures and Tables

**Table 1:** Percentage of non-susceptible isolates detected in bird droppings, according to experimental groups after oxytetracycline treatment.

Experimental Groups	Strains	Post-Treatment Days					*p*-Value ^1^
		0	1	7	14	21	
Treated	Total number of isolates	16	20	19	22	23	
	Non-susceptible strains (%)	37.5	100	84.21	63.63	34.8	
	Susceptible strains (%)	62.5	0.0	15.78	36.36	65.2	3.5 × 10^−7^
Untreated	Total number of isolates	30	13	17	12	14	
	Non-susceptible strains (%)	0	76.92	41.17	8.33	28.57	
	Susceptible strains (%)	100	23.07	58.82	91.66	71.42	

^1^ Significance difference *p* < 0.05 (chi-squared test).

**Table 2:** Percentage of non-susceptible isolates detected in poultry litter, according to experimental groups after oxytetracycline treatment.

Experimental Groups	Strains	Post-Treatment Days						*p*-Value ^1^
		1	7	14	21	29	36	
Treated	Total number of isolates	29.0	12.0	18.0	17.0	22.0	25.0	
	Non-susceptible strains (%)	82.8	75.0	72.2	17.6	68.2	92.0	
	Susceptible strains (%)	17.2	25.0	27.8	82.4	31.8	8.0	2.2 × 10^−16^
Untreated	Total number of isolates	52	15	15	10	10	25	
	Non-susceptible strains (%)	3.8	0.0	0.0	30.0	30.0	8.0	
	Susceptible strains (%)	96.2	100.0	100.0	70.0	70.0	92.0	

^1^ Significance difference *p* < 0.05 (chi-squared test).

**Table 3:** Primers and conditions for each tetracycline resistance gene.

Gene	Sequence (5′ > 3′)	Annealing Temperature (°C)	Size	Reference
*tet*(A)	F: GCTACATCCTGCTTGCCTTC	54	210	[46]
	R: CATAGATCGCCGTGAAGAGG			
*tet*(B)	F: TTGGTTAGGGGCAAGTTTTG	54	659	[46]
	R: GTAATGGGCCAATAACACCG			
*tet*(C)	F: CTTGAGAGCCTTCAACCCAG	54	418	[46]
	R: ATGGTCGTCATCTACCTGCC			
*tet*(D)	F: AAACCATTACGGCATTCTGC	54	787	[46]
	R: GACCGGATACACCATCCATC			
*tet*(E)	F: AAACCACATCCTCCATACGC	54	278	[46]
	R: AAATAGGCCACAACCGTCAG			
*tet*(M)	F: GTGGACAAAGGTACAACGAG	54	406	[46]
	R: CGGTAAAGTTCGTCACACAC			
*16s*RNA	F: GACCTCGGTTTAGTTCACAGA	54	585	[47]
	R: CACACGCTGACGCTGACCA			
*E. coli uspA*	F: CCGATACGCTGCCAATCAGT	54	884	[43]
	R: ACGCAGACCGTAGGCCAGAT			

## Data Availability

The data presented in this study are available in the article.
